# Acceleration in Germination *Sensu stricto* Plays a Central Role on Seedling Vigor in Post-Germination

**DOI:** 10.3390/plants10102151

**Published:** 2021-10-11

**Authors:** João Paulo Ribeiro-Oliveira, Marco Aurélio Bosseli, Edvaldo Aparecido Amaral da Silva

**Affiliations:** 1 Instituto de Ciências Agrárias, Universidade Federal de Uberlândia, Santa Mônica, Uberlândia 38408-100, MG, Brazil; 2 Instituto de Física, Universidade Federal de Uberlândia, Santa Mônica, Uberlândia 38400-902, MG, Brazil; maboselli@gmail.com; 3 Department of Crop Science, College of Agricultural Science, São Paulo State University, José Barbosa de Barros Street, 1780, Botucatu 18610-307, SP, Brazil; amaral.silva@unesp.br

**Keywords:** acceleration, imbibition, biological activity, indexes, germination measurements, seed physiology, soybean, velocity

## Abstract

An obvious relationship between germination *sensu stricto* and seedling development during post-germination has been considered, but not explained concerning vigor. Taking this into account, we used measurements of water dynamics in germinating seeds and seedling development to clarify that relationship. The biological model was soybean seeds, since it is the most relevant ‘true seed’ produced around world. Our findings suggest that the way energy is used (acceleration) and not its input (velocity) is the main aspect relating seed germination and seedling development, especially when considering vigor. However, velocity and acceleration can be complementary in analyses of seed physiology. Other measurements proposed here also have potential uses for testing vigor in seed lots, such as seedling vigor index and biological activity in the lot. Therefore, water dynamics in germinating seeds can be an interesting way for testing seed lots, because it is an easier, faster and cheaper method in relation to other non-destructive procedures.

## 1. Introduction

Vigor is a functional trait acquired in the last stages of seed maturation and is responsible for seed resilience. In this sense, it is expected that vigorous seeds should possess more capacity to produce early seedlings [1]. This in turn reduces the possibility of their suffering with inter- and intra-specific competition during early plant development. Because of that, this idea has been explored by basic and technical science to predict the behavior of seed germination and plant establishment in field conditions.

Even though the impact on cultivation caused by seed vigor differs among species, depending on specific production practices [2,3], considerable reductions in yield might be expected when non-viguorous seeds are sown. As an example, over the past few decades, there have been reports indicating reductions of up to 50% in yield components of soybean due to the use of non-vigorous seeds [4,5]. This justifies continuous research to provide practical, rapid and simple ways of predicting seed-lot vigor. By the way, not surprisingly, most information on seed-lot vigor is based on early plant development, especially on seedling development, which is the basis of national and international seed trade [6,7]. However, details about interactions between germination *sensu stricto* [1 sense] and seedling vigor (immediate post-germination) still have to be clarified. Thus, we asked: what is the relationship between germination *sensu stricto* and seedling development of commercial lots? The answer can not only be used to promote a new and rapid test of seed vigor, but can also be a bridge between seed physiology and seed technology. Recent studies consider this bridge to be water dynamics in germinating seeds [2,8,9].

Water dynamics in germinating seeds has been used as a trivial laboratory test, which is incorrectly called imbibition test [9]. However, studies focusing on this relation dating back to the early 1980s demonstrate that the entrance and exit of water in a live cell is an important physiological trait of germination *sensu stricto* because of the integration with biophysical and/or biochemical processes [10,11,12,13,14]. In this occasion, although some authors explored the kinetic idea for water influx in germinating seeds, vigor in germination *sensu stricto* was poorly explored [12]. In previous years, a physiological sense was offered to define seed vigor, by considering it a characteristic related to the embryo’s ability to perform the germination process in a coordinated and sequential way [2]. In this sense, new tests of water dynamics in germinating seeds were based on infrared and/or magnetic resonance [15,16,17,18,19]. These tests are useful, but still expensive and therefore not always possible to be used routinely in seed testing. To make use of the phenomena in an easy and practical way, water dynamics measurements were proposed [9,20]. More specifically, velocity and acceleration were proposed as a tool to investigate germination *sensu stricto* in the seed-seedling transition [21]. Velocity is a functional trait for metabolism since it describes the variation of mass over germination time, whereas acceleration measures the variation of velocity. However, no study has been done to understand the relationship between measurements of water dynamics in germinating seeds and seedling development in immediate post-germination. Thus, our hypothesis is that velocity and/or acceleration of water dynamics in germinating seeds can be a biomarker of vigor, improving the knowledge about how this influences the seedling development of a commercial lot. We also expand on how measurements of water dynamics can be used in a physiological sense.

## 2. Material and Methods

### 2.1. Biological Model

Soybean was used as a biological model in this paper, since (i) the species is the world’s largest source of animal protein feed and the second largest source of vegetable oil [22], being the number one ‘true seed’ in cultivated areas of the world (only maize, wheat and rice, which have caryopses, are more cultivated); (ii) it is the agricultural product with the highest commercial growth rate in world [23]; and (iii) it has classical reports related to water dynamics in germinating seeds [13,14]. Soybean is also one of the most important genetically engineered (GE) crops, the main type of cultivation in traditional grain producers as the United States, Argentina and Brazil [24]. Taking this into account, seeds of NA5909RR cultivar transgenic soybean (*Glycine max* L.) were used, with an initial moisture content of 11%. The cultivar is one of the most cultivated in Brazil because of its high adaptability in diverse edafic-climatic conditions. 

A seed private company provided the seed lots (produced according to cultivation and harvesting procedures defined by the protocol of the donor) without any chemical treatment. These seeds were produced in the 2013–2014 Brazilian crop season. At first, we made a screening of 15 lots. The physiological quality of the lots was evaluated in pre-testing, quantifying germinability, viability and germination initial time. From this, we defined three samples (with low, intermediate and high quality). The designations low, intermediate and high follow the ones proposed by Ribeiro–OliveiraandRanal [9,25], who defined them based on viability and germination measurements. Samples composed by seeds with viability (*V*) and germinability (*G*) higher than 90% were considered high-quality, whereas *V* ≤ 60% and *G* ≤ 50% were considered low-quality. The seeds with intermediate quality had intermediate values for both characteristics. This also allowed us to comply with the International Seed Testing Association (ISTA) methods for seed-testing validation [26]. The ISTA, based on ISO5725-2 [27], determines that any method of germination analysis can only be validated when using samples with different physiological standards. We highlight that these inferior boundaries are not considered a seed sample in a seed technology context, but they are important for plant breeders and seed physiologists who study samples of lineages and/or other plant species with germinability and viability below the soybean-seed trade standards. 

### 2.2. Water Dynamics in Germinating Seeds

Seeds were sowed in germination boxes (plastic boxes) over paper soaked with distilled water (volume in mL equivalent to six times the mass of the paper in g) and then placed on a laboratory bench at 25.3 ± 1.5 °C under white fluorescent light (11.29 ± 2.84 µmol m^−2^ s^−1^ Photosynthetic Photon Flux Density—PPFD). The initial water volume (lost either by experimental manipulation or possible evaporation) was maintained by adding 1 mL (defined in pre-testing) of distilled water in the germination box after each mass recording. These seeds (*n* = 50; more than sufficient sample size for this test according to 9) were individually weighed on a digital scale (at 0.0001 g precision) every hour until 2 h after embryo protrusion (root or cotyledon emergence). The germination boxes were opened just for the mass recording of the seed individual, assuring the germination boxes would have characteristics similar to a humidity chamber (high vapor pressure). The water dynamics in germinating seeds was measured by means of this recording of mass over time. As this is associated with germination *sensu stricto*, measurements of water dynamics in germinating seeds are functional traits related to the embryo.

#### Modelling the Water Dynamics in Germinating Seeds

For modelling the water dynamics in germinating soybean seeds, we used the algorithm proposed by Ribeiro-Oliveira et al. [21]. Therefore, the weighted mass of the water dynamics in germinating seeds was obtained from mass data collected over time and weighted by initial mass (hydroscopic equilibrium). Raw data of mass were used to calculate the coefficient of initial diffusion, which is a function of the water diffusion ratio by the seed radius (*D*/ρ^2^; *n* = 50). This ratio was used focusing on a practical sense, since we used seeds with similar dimensions (Φ = 6.9 ± 0.3; mean ± standard error) for each sample. We parameterized *D*/ρ^2^ since errors from approximations from seed shapes are partially compensated [28]. We used the diffusion coefficient per unit area as an initial diffusion coefficient because it makes the parameter comparable for species or samples with different seed sizes. We also used the bootstrap method with 1000 re-samplings, since values generated above this number are similar according to the convergence test, as an assumption to calculate weighted mass and, then, means of velocity and acceleration. To calculate these confidence intervals, the Algorithm AS 214 for Fortran was used [29], which is recommended to perform the Monte Carlo Confidence Intervals. The central point of these measurements and the base of the quantitative treatment is m, the water mass over time. The tabulated values of the normalized mass $m\left(t\right)$ were obtained from total mass $M\left(t\right)$ divided by initial (dry) seed mass ($M_{0}$).

The first step in the numerical calculation is the interpolation of $m\left(t\right)$ by cubic splines [30], and as the main characteristic of this method is the interpolated function, its first and second derivatives are continuous. The result is a smooth function that can be used to study metabolic changes as a function of time. For details see Ribeiro-Oliveira et al. [21].

Velocity is expressed as

(1) $$v=\frac{dm}{dt}$$

and acceleration in our analyses will be

(2) $$a=\frac{d^{2}m}{dt^{2}}$$

Within the interpolation scheme, it is simple to integrate the data from Equations (1) and (2), and calculate the average values $v_{m}$ and $a_{m}$ for velocity and acceleration, respectively. It is worthwhile mentioning that velocity is associated to the flux of water from the outside to the inside of the seed. The time average $a_{m}$ of a parameter $a\left(t\right)$ is defined by

(3) $$a_{m}=\frac{1}{\tau }\int_{0}^{\tau }a\left(t\right)dt$$

where τ is the last time tabulated for each sample. The definition (3) is also used to calculate $v_{m}$.

The diffusion ratio coefficient defined by $D/\rho^{2}$ and the saturation mass $M_{\infty }$ were fitted for a series solution of the Fick differential equation for a spherical model of the seed through the expression (4)

(4) $$\frac{M\left(t\right)}{M_{\infty }}=1-\frac{6}{\pi^{2}}\overset{\infty }{\underset{n=1}{\sum }}\frac{1}{n^{2}}exp\left(-Dn^{2}\pi^{2}t/\rho^{2}\right)$$

where $M\left(t\right)$ is the total mass of the diaspore at a time *t*, D is the diffusion coefficient, ρ is the radius of the seed, *t* is the time, *n* is the number of terms in the series. The best fit in a sequence with different values defines the choice of *n*. The values of $D/\rho^{2}$ and $M_{\infty }$ were obtained by a nonlinear fitting, using the Levenberg–Marquardt method [31]. These parameters were fitted using only data from the first hours of the experiment, limiting them to the purely imbibition phase, just before the presence of water trigger biochemical processes in the seed. $M\left(t\right)$ in the Equation (4) is the total mass, to account for the boundary conditions imposed by the Equation correctly (4).

### 2.3. Seed Germination Assays

We maintained the seeds used in the water dynamics assessment for 48 h in the same experimental conditions to analyze them in relation to germinability (*G*; percentage of germination) and viability (*V*). Viability was calculated using the proportion of viable seeds (evaluated by means of 2,3,5-triphenyl-2H-tetrazolium chloride solution—TTC) in relation to the total seeds analyzed [32]. 

From the data of germinability and viability, we calculated Biological Activity (*A*) in the sample. *A* is a measurement classically used as an analogous to the equation relating the thermodynamic activity of a solute to its concentration via an activity coefficient [33]. The activity is related to the capacity of a biological system to manage chemical processes (endogen and exogen), which is similar to the idea of seed germination in a sample. For that, the expression considers the amount-of-substance concentration or, in this case, proportion of germinated seeds (*c*) and a parameter designated as inherent activity, i.e., here, the proportion of viable seeds in the sample (*f*). We are transposing this concept to seed science by using an adaptation in the algebraic expression (5):

(5) A=G V 100

### 2.4. Seedling Development Assays

The seedling development assays were performed in germination chamber from a completely randomized design (CRD). The chamber was configured under continuous white fluorescent light (15.79 ± 3.70 µmol m^−2^ s^−1^ PPFD) at 25.0 ± 2.7 °C. We performed the sowing in layers of germination paper moistened with distilled water in the proportion of 2.5 times the dry mass of the paper in milliliters [32]. We used a sample (*n*) with 200 seeds (*n* = 200). The seedlings were observed and classified on the eighth day after sowing [32]. They were classified as normal seedlings, damaged and deteriorated abnormal seedlings, and dead or dormant seeds [32].

The seedling development protocol was also the basis for the experimental design of the accelerated ageing test (*AA*), one of the most used seed vigor tests in the world for several species [34], which is recommended for soybean seed testing [35]. We used this test to determine seedling vigor in place of other classical measurements, such as relative growth rate, because our interest is to connect seed physiology to seed technology. Thus, for the AA test, the seeds were exposed to non-optimum relative humidity and temperature {41 °C ± 0.3 °C and ~95% humidity for 72 h ± 15 min, according to [35]} before sowing in germination paper, to generate a stress condition for the embryo. On the fifth day after sowing, the number of normal seedlings was determined, and their percentage in relation to the number of seeds sowed was expressed as the test result.

In addition to the *AA* test, seedling vigor was examined by means of two other characteristics, seedling vigor index (*SVI*) and seed-seedling transition yield index (*SSYI*). Both indexes are based on the ability of a seed to produce a normal seedling. The *SVI* is based on the vigor index proposed by [36] and modified by [37]. The authors also used the *AA* and seedling emergence results to define a measurement for seedling vigor of soybean seeds. However, as our purpose was to analyze how germination *sensu stricto* is correlated to seedling vigor, we performed an adaptation, since it is only possible to correlate characteristics obtained in similar experimental conditions [38]. Taking this into account, we adapted the vigor index to seedling vigor index by using the algebraic expression (6):

(6) $$SVI=\frac{AA}{NS}100$$

where: *SVI* is the seedling vigor index, expressed in percentage, *NS* is the proportion of normal seedlings developed from non-stressed seeds, and *AA* is the proportion of normal seedlings developed from stressed seeds exposed to the accelerated ageing test. By taking only normal seedlings from each condition into account [39], the measurement can be more interesting to predict the seedling vigor in a *sensu stricto* view. Apart from that, we used a simple index based on the yield idea to observe how many seeds in the sample were able to develop a normal seedling. For that, we used the following algebraic expression (7):

(7) $$SSYI=\frac{NS}{G}100$$

where SSYI is the seed-seedling transition yield index, expressed in percentage, *NS* is the percentage of normal seedlings developed from non-stressed seeds and in an optimum experimental condition, and *G* is the germinability of the sample in an optimum experimental condition.

### 2.5. Statistical Analysis

For water dynamics in germinating seeds, confidence intervals at 0.05 significance were calculated for the mean values by means of Algorithm AS 214 for Fortran [30], which is recommended to perform the Monte Carlo Confidence Intervals. Overlapping confidence intervals indicate non-significant differences [32]. For seed germination and seedling measurements, we adopted the Binomial distribution with logit function (from glm2 package in R project; https://cran.r-project.org/, accessed on 7 September 2021); every zero value was observed. Means comparisons were performed using the Tukey test and by using confidence intervals calculated by the Šidák correction to reduce the familywise error rate–FWER [40]. In these cases, we also used at 0.05 significance (α = 0.05). In addition, we calculated the Pearson linear correlation between the water dynamics in germinating seeds, seed germination (embryo protrusion), seed viability and post-germination (seedling development) measurements by using the residuals to standardize the effect of samples, as was recommended by [38]. The *r* values were tested by the Student *t* test at 0.01 significance (α = 0.01), and only characteristics with differences were used to build a heatmap from script defined by [41]. The adjectives to describe the magnitude of the correlations were proposed by [42], where the values from *r* = 0.01 to 0.09 are negligible correlations, *r* = 0.10 to 0.29 are low, *r* = 0.30 to 0.49 are moderate, *r* = 0.50 to 0.69 are substantial, *r* = 0.70 to 0.99 are very high, and *r* = 1.0 is the perfect correlation.

## 3. Results

**Figure 1:**
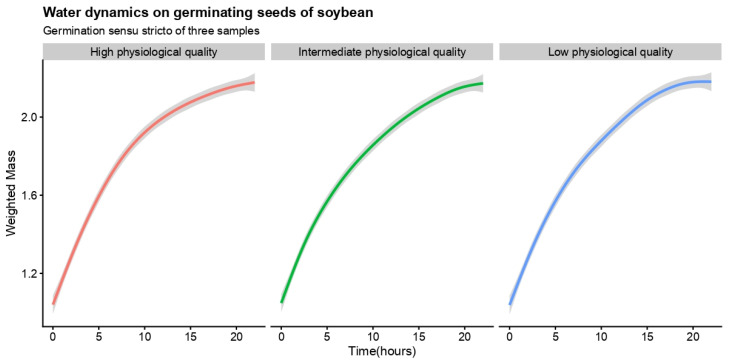
Water dynamics in germinating seeds of soybean based on weighted mass over time. Physiological quality describes viability (*V*) and germinability (*G*) in the sample from pre-testing: High physiological quality is related to a seed sample with viability (*V*) and germinability (*G*) higher than 90%, intermediate physiological quality is a seed sample with 61% ≤ *V* ≤ 89%9 and 51% ≤ *G* ≤ 89%, and low physiological quality is a seed sample with *V* ≤ 60% and *G* ≤ 50%. The solid line (red, green and blue) shows the mean value, and the gray area delimits the lower and upper confidence intervals from 1000 Monte Carlo simulations at 0.05. The embryo protrusion in at least one sample occurred 2 h before the last recording. *n* = 50 diaspores.

The water dynamics in germinating seeds of the three samples based on weighted mass over time (germination *sensu stricto*) were shown to be similar (Figure 1). The seed samples had three behaviors in relation to germinability and viability (Table 1), ratifying the pre-testing. These behaviors were also observed regarding the capacity of seed-seedling transition (Table 1), being the sample previously categorized as high physiological quality with a high ability to produce normal seedlings (*NS* = 98.00%). All seeds germinated in this sample developed a normal seedling, reaching 100% in the seed-seedling transition yield index (*SSYI*). In addition, the high-quality seed sample, with 99.94% of biological activity (*A*; Table 1), developed lower abnormal seedlings and dead seeds in the sample, both at about 1% (Table 1). These values were coherent with the high resilience of seeds of the sample, developing 94% normal seedlings after accelerated ageing (Table 1). Besides seed resilience, the sample also demonstrated a high capacity to develop vigorous seedlings (*SVI* = 95.92%). 

The lower the physiological seed quality in a sample, the lower the biological activity (Table 1). This led to 24% and 48% normal seedlings developed from seeds of low- and intermediate-quality samples, respectively (Table 1). Seeds that failed to germinate were more relevant to physiological designations than seedling growth, since although the number of abnormal seedlings was similar between low (*AS* = 25%) and intermediate (*AS* = 27%) physiological samples, the number of dead seeds in a low-quality sample was approximately twice as large when compared to an intermediate quality sample (Table 1). Although seed resilience to non-optimum relative humidity and temperature is lower in low-physiological seeds (*AA* = 16%) than in intermediate-physiological seeds (*AA* = 34%), the seedlings developed from these seeds had a similar vigor to those originated from seeds of intermediate quality (77.27% ≤ seedling vigor index ≤ 80.00%).

No sample had seeds with physical dormancy [42] (0% of hard seeds), enabling practical inferences about germination *sensu stricto* from measurements of water dynamics in germinating seeds (Table 1). As detected by the accelerated ageing test, the initial diffusion coefficient also demonstrated three distinct behaviors of seeds from the samples (Table 1). The higher initial diffusion coefficient ($D/\rho^{2}$ = 0.0014 h^−1^) of water in germinating seeds of soybean was observed in those from the intermediate physiological quality sample ($D/\rho^{2}$ = 0.0018 h^−1^). These seeds also had the highest values of mean velocity ($v_{m}$ = 0.058 g H_2_O h^−1^) of water dynamics during germination *sensu stricto* (Table 1). On the other hand, the highest values of mean acceleration of water dynamics were obtained for seeds from the high-quality sample ($a_{m}$ = −0.0164 g H_2_O g^−2^; Table 1). 

**Figure 2:**
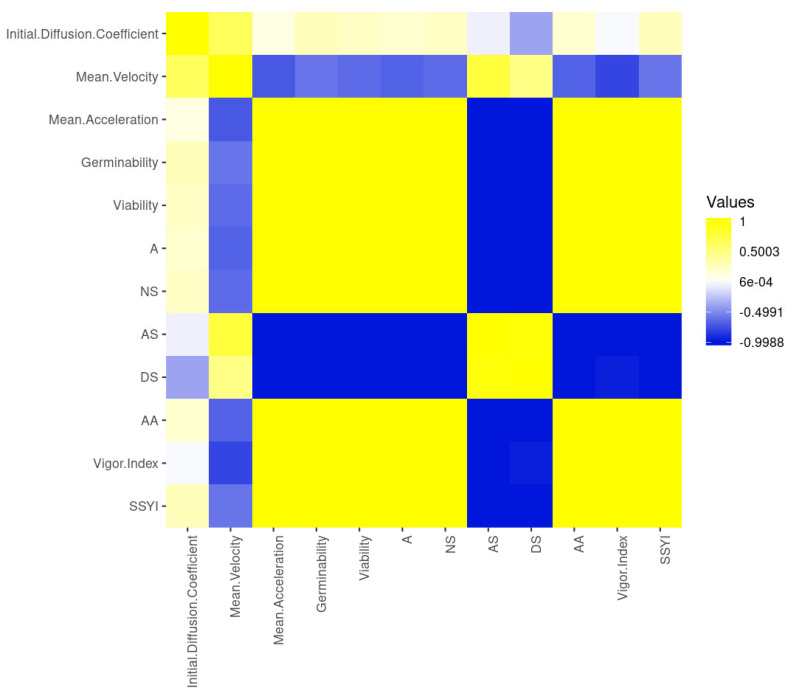
Correlation matrix heatmap based on pair-wise Pearson correlation coefficients (rank correlation; *p* ≤ 0.01) for germination *sensu stricto* and post-germination (seedling development) measurements of soybean seeds. Initial diffusion coefficient: $D/\rho^{2},$ h^−1^; mean velocity: $v_{m}$, g H_2_O h^−1^; mean acceleration: $a_{m},$ g H_2_O h^−2^; *G*: germinability (%); *V*: viability (%); *A*: Biological Activity in the sample (%); *NS*: Normal seedlings (%); *AS*: Abnormal seedlings (%); *DS*: dead seeds (%); *AA*: percentage of normal seedlings after accelerate ageing test; Vigor index: *SVI* = Seedling vigor index (%); *SSYI*: seed-seedling transition yield index (%).

The early steps of germination *sensu stricto* affected the velocity positively and linearly as the germination occurred, but they did not impact acceleration (Figure 2). This was proved by the perfect [43] linear correlation to the initial diffusion coefficient and mean velocity of water dynamics in germinating seeds, and moderate linear correlation to former measurements and mean acceleration (Figure 2). From these measurements, only mean acceleration had a positive, linear and substantial correlation to seedling development (post-germination), observed by correlations among seed germinability, viability and normal seedlings developed. In addition, mean acceleration of water dynamics in germinating seeds had a positive and substantial linear correlation to the seedling vigor index, *SSYI*, and *A*; whereas, mean acceleration was substantially negatively correlated to abnormal seedlings developed and the number of dead seeds in a sample (Figure 2). This demonstrates that mean acceleration is associated to both embryo and seedling vigor. By the way, germinability, viability, biological activity, normal seedlings developed; seedling vigor index and *SSYI* had substantial positive linear correlation among themselves; and there was a substantial negative correlation to abnormal seedlings development and dead seeds (Figure 2). What draws our attention, on the one hand, is the low and negative linear correlation between mean velocity and the seedling vigor index as well as between initial diffusion coefficient and dead seeds; on the other hand, it is also interesting to note the low and positive linear correlation between mean velocity and abnormal seedlings developed. The seedling vigor index also had a negligible negative linear correlation to dead seeds, whereas the initial diffusion coefficient had a negligible positive linear correlation to all measurements related to protrusion (germinability, viability and biological activity), normal seedlings (from seeds exposed or not to stress) and *SSYI* (Figure 2). These results not only demonstrate that the initial diffusion coefficient is an interesting measurement to infer germination, but they also mean that velocity and mean acceleration of water dynamics in germinating seeds are complementary to analyses about seed physiology and immediate post-germination.

## 4. Discussion

Our findings demonstrate that although germination *sensu stricto* and seedling vigor related to post-germination do not always have the same contribution to seedling development, there are traits of water dynamics in germinating seeds capable of building a bridge between them. Specifically, the mean acceleration of water dynamics in germinating seeds is a measurement related to both germination *sensu stricto* and post-germination, including the development of vigorous normal seedlings. For the first time, we prove the relevance of acceleration in seed germination for seed-seedling transition. Up to now, only velocity has been considered as an important aspect for this transition. However, with a new model for the germination process, based on biophysics measurements and with robustness to predict metabolism, we amplify the concept around the seed-seedling transition in a context of energy management by means of acceleration. 

Other methods have been established as a bridge between germination *sensu stricto* and seedling development, such as biospeckle in coffee seeds [44]. The fact is: as the transition zone hypocotyl-radicle elongates during germination *sensu stricto* [45,46], correlations between a biophysical signal and cell elongation demonstrate that it is possible to make inferences of seedling development from a germinating seed [44]. What we offer here is a safer and cheaper way to do it, by using parameters from water dynamics in germinating seeds from a simple mass measurement over time. These measurements were recently proposed by our group to demonstrate phases of germination and post-germination, as well as to prove that the ‘three-phase germination model’ is not a standard, but an exception [21].

Mean acceleration and mean velocity were applied here by means of knowledge from biophysics. According to our assumption, better functioning and metabolism during germination is accompanied by greater velocity and acceleration. This relationship is expected because velocity is directly associated with water flux and, consequently, is a measurement of the variation of both diffusion (especially during imbibition per se, respecting Fick’s law) and catabolism and anabolism [21]. In addition, acceleration is a rate of velocity change and, therefore, could reflect metabolism more accurately. Here, our assumption is validated for the seed-seedling transition since the mean acceleration was highly and positively correlated to seedling vigor. 

Biological activity is a principle of pharmacology in which the greater the presence of an active principle in a living system, the more biological activity it has [33,47,48]. This activity is mainly dependent on the uptake, distribution and metabolism of energy by the living system. It is important to note that metabolism is defined by velocity and acceleration. Velocity is considered an energy input measurement, whereas acceleration is an energy use measurement [21]. This is naturally related to chemical kinetics, but needs to be considered in more complex models such as that defined by Onsager [49]. In seed science, only mean velocity has been used [50], and it was recently considered a species-specific trait [51]. This is coherent with our findings, since only intermediate-quality seeds demonstrated a different mean velocity. This sample was obtained by applying thermal stress to seeds in anhydrobiosis. Stress conditions have the potential to repress species-specific traits because of selective pressure, leading to a possible segregation in the population [52,53,54]. In addition, it is expected that extreme differences in physiological quality of seeds of the same sample will promote, on the one hand, faster and non-controlled water dynamics, and, on the other hand, slower and controlled water dynamics in germinating seeds [2,4,55]. This can be considered a limitation for using mean velocity. However, when we overlapped it with acceleration, this limitation was overcome. By inferring energy use, mean acceleration is expected to be more related to embryo vigor than to mean velocity. Because of that, low- and intermediate-physiological quality seeds possess similar mean acceleration, which is lower than in high-quality seeds. The low- and intermediate-physiological quality samples had a similar seedling vigor index since the seedlings from the sample had similar development. However, the intermediate-physiological quality seeds had a greater ability to transit from seed to seedling than low-quality ones because they possessed more biological activity. This was corroborated by the correlation analysis, and it also explains why the intermediate-physiological quality sample had more resilience to the accelerated ageing test than the low-quality sample. 

As might be expected [56], low- and intermediate-physiological quality seeds stand out in imbibition per se due to high intraspecific variability (see parameters and confidence intervals in Table 1). That occurred with soybean seeds, which have a slightly thick tegument and a protein reserve, due to peculiarities in membrane rehydration and high affinity of protein to water. As low-quality seeds have high cellular unviability (low viability), there is a failure in membrane selectivity, which controls water influx and inner cell maintenance, especially in the imbibition phase [55,57], making the initial diffusion in low-quality seeds faster than in high-quality seeds. In intermediate-quality seeds, membrane control occurs, but not as efficiently as in high-quality seeds, increasing intraspecific differences observed by CI and velocity of imbibition. Due to that, the initial diffusion coefficient is correlated to metabolic (mean velocity and mean acceleration) and physiological (germinability, viability and biological activity) measurements of embryo protrusion and, therefore, can infer seedling vigor. This also demonstrates that the initial diffusion coefficient must be able to infer physical and physiological quality in a seed sample, especially soybean. After all, the more damaged a tegument is, the lower the membrane control is in the imbibition process [9,10,14,19]. However, this can be studied in the future by using a more adequate experimental design.

The explanation about the supposed incoherence between *SSYI* and the seedling vigor index is algebraic and physiological. In the algebraic sense, *SSYI* is a simple index of yield, based only on the quantity of germinated seeds that grow and get normal seedling status, whereas the seedling vigor index is based on the number of normal seedlings developed from stressed and non-stressed seeds, in this case simulated by the ageing acceleration test. In all cases, *SSYI* and the seedling vigor index are measurements defined from viable seeds, whereas the results of *AA* consider both viable and non-viable seeds in its calculation. Therefore, *SSYI* and seedling vigor index are weighted characteristics, which enable inferences on seedling vigor *sensu stricto*. Contrary to this, *AA* offers an estimative on seed sample vigor based on the resilience behavior of seeds in non-optimum humidity and temperature [6,28,54]. This is more objective by considering the physiological sense from the General Adaptation Syndrome of Hans (János) Selye [52,58]. From that, stressed seeds have three stages before death. First, they recognize the environmental cues as ‘priming’ (alarm phase). If the stress persists, the seeds trigger molecular defenses based on species-specific adjustments, which can lead to resilience (resistance phase) or a degradation of biological functions (exhaustion phase). A consequence is an increase of abnormal seedlings and/or dead seeds, which is expected as one of the last events in seed deterioration (both natural and artificial) [52]. This occurred here and made seedlings that developed from the low-quality sample have similar vigor to the ones developed from the intermediate-quality sample. This led to a similar ability in germinated seeds to transition to seedling in both samples, but with differences in quality of the samples when considering the number of seeds in each one. 

Several authors have been trying to establish a relationship between functional traits of germination *sensu stricto* and post-germination [8,18,59], but few got representative results about seedling vigor [2,44,45]. Nowadays, magnetic resonance has built a bridge between those two phenomena [15,16,17,19] but this technique is both expensive and specialized, requiring an advanced laboratory. Here, we are presenting not only relations between these steps, but also demonstrating that water dynamics is associated with both embryo vigor and seedling vigor. This can be useful to predict vigor in seed samples in routine and academic laboratories in an easier, cheaper and more practical way in the near future. For example, image analyses can be useful (such as those based on ImageJ®; https://imagej.nih.gov/ij/, accessed on 7 September 2021). Several software applications have been presented to the seed industry that can be updated for analysis of germination *sensu stricto* to predict seedling vigor. Taking this into account, this paper can serve as a guideline. In any case, other studies must be designed, especially for other species with dormant seeds. In the end, the findings suggest that embryo vigor can influence seedling vigor and, therefore, seed sample vigor, because the energy used by the embryo during germination *sensu stricto* is fundamental to early plant development. This directly affects the resilience of a commercial sample to stresses and, consequently, its ability to produce normal seedlings. It is important to note that the kinetics of germination *sensu stricto* is not very representative of vigor, which may come as a surprise to seed technologists. However, again, an addendum is that in seed technology, the germination *sensu stricto* could be considerate secondary; for this science, the kinetics observed are about seedling development, which can justify velocity as an important aspect of vigor. Here, we are presenting a way of speeding the testing of vigor seed for both seed physiologists and seed technologists, by associating both processes of early plant development. In this sense, mean acceleration of the water dynamics in germinating seeds can be an important measurement to demonstrate how germination *sensu stricto* affects the seedling vigor positively.

## Figures and Tables

**Table 1:** Seed-seedling transition measurements in soybean (*Glycine max* L.).

Seed-Seedling Transition Step	Seed Sample	$D/\rho^{2}(h^{-1})\;$	$v_{m}\;(g\;H_{2}O\;h^{-1})$	$a_{m}\;(g\;H_{2}O\;h^{-2})$			
Germination *sensu stricto*	Low physiological quality	0.0014	0.055 (0.0279; 0.0822)	−0.0184 (−0.0391; 0.0298)			
	Intermediate physiological quality	0.0018	0.058 (0.0278; 0.0882)	−0.0182 (−0.0468; 0.0105)			
	High physiological quality	0.0016	0.055 (0.0355; 0.0074)	−0.0164 (−0.0310; −0.0019)			
Embryo protrusion		*G* (%)	*V* (%)	*A* (%)			
	Low physiological quality	28.00 ± 3.8 c	48.00 ± 4.2 c	26.36 ± 0.36 c			
	Intermediate physiological quality	54.00 ± 4.2 b	62.00 ± 4.1 b	65.66 ± 0.39 b			
	High physiological quality	98.00 ± 1.2 a	98.00 ± 1.2 a	99.94 ± 0.02 a			
Post-germination (seedling development)		*NS* (%)	*AS* (%)	*DS* (%)	*AA* (%)	*SVI* (%)	*SSYI* (%)
	Low physiological quality	24.00 ± 3.4 c	25.00 ± 0.008 b	51.00 ± 0.008 c	16.00 ± 3.7 c	80.00 ± 3.6 b	71.43 ± 3.81 c
	Intermediate physiological quality	48.00 ± 4.0 b	27.00 ± 3.61 b	23.00 ± 3.66 b	34.00 ± 4.0 b	77.27 ± 3.4 b	81.48 ± 3.29 b
	High physiological quality	98.00 ± 1.0 a	1.00 ± 3.7 a	1.00 ± 4.21 a	94.00 ± 2.0 a	95.92 ± 1.7 a	100.00 ± 0.01 a

Physiological quality describes viability (*V*) and germinability (*G*) in the sample from pre-testing: High physiological quality is related to a seed sample with viability (*V*) and germinability (*G*) higher than 90%, intermediate physiological quality is a seed sample with 61% ≤ *V* ≤ 89% and 51% ≤ *G* ≤ 89%, and low physiological quality is a seed sample with *V* ≤ 60% and *G* ≤ 50%. *A:* biological activity in the sample; *D*/*ρ*^2^: Initial diffusion coefficient of water dynamics in germinating seeds; $v_{m}$: mean velocity of water dynamics in germinating seeds; $a_{m}$: mean acceleration of water dynamics in germinating seeds; *NS*: percentage of normal seedlings; *AS*: percentage of abnormal seedlings; *DS*: dead seeds; *AA*: percentage of normal seedlings after accelerated ageing test; *SVI*: Seedling vigor index; *SSYI*: seed-seedling transition yield index. *D*/*ρ*^2^ is a parameter of Fick’s Second Law from a data set to perfect fitting and, hence, without statistical dispersion measurements; only absolute values. The values of germination *sensu stricto* measurements represent the mean (lower confidence interval; upper confidence interval) obtained from 1000 Monte Carlo simulations at 0.05. For each measurement of embryo protrusion and/or post-germination measurements, means and confidence intervals of the Šidák method followed by different letters are significantly different (*p* < 0.05) as compared using a Tukey test.

## Data Availability

The data presented in this study are available on request from the corresponding author. The data are not publicly available due to founding restrictions.
